# Antimicrobial Resistance in *Escherichia coli* Recovered from Feedlot Cattle and Associations with Antimicrobial Use

**DOI:** 10.1371/journal.pone.0143995

**Published:** 2015-12-03

**Authors:** Katharine M. Benedict, Sheryl P. Gow, Tim A. McAllister, Calvin W. Booker, Sherry J. Hannon, Sylvia L. Checkley, Noelle R. Noyes, Paul S. Morley

**Affiliations:** 1 Department of Clinical Sciences, College of Veterinary Medicine and Biomedical Sciences, Colorado State University, Fort Collins, Colorado, United States of America; 2 Laboratory for Foodborne Zoonoses, Public Health Agency of Canada, University of Saskatchewan, Saskatoon, Saskatchewan, Canada; 3 Lethbridge Research Center, University of Lethbridge, Lethbridge, Alberta, Canada; 4 Feedlot Health Management Services, Ltd., Okotoks, Alberta, Canada; 5 Faculty of Veterinary Medicine, University of Calgary, Calgary, Alberta, Canada; U. S. Salinity Lab, UNITED STATES

## Abstract

The objectives of this study were to estimate the prevalence of antimicrobial resistance (AMR) and to investigate the associations between exposures to antimicrobial drugs (AMDs) and AMR in fecal non-type specific *Escherichia coli* (NTSEC) recovered from a large population of feedlot cattle. Two-stage random sampling was used to select individually identified cattle for enrollment, which were sampled at arrival and then a second time later in the feeding period. Advanced regression techniques were used to estimate resistance prevalences, and to investigate associations between AMD exposures in enrolled cattle and penmates and AMR identified in NTSEC recovered from the second sample set. Resistance was most commonly detected to tetracycline, streptomycin, and sulfisoxazole, and was rarely identified for critically important AMDs. All cattle were exposed to AMDs in feed, and 45% were treated parenterally. While resistance prevalence generally increased during the feeding period, most AMD exposures were not significantly associated with AMR outcomes. Exposures of enrolled cattle to tetracycline were associated with increased resistance to tetracycline and trimethoprim sulfa, while beta-lactam exposures were associated with decreased likelihood of detecting streptomycin resistance. Pen-level AMD exposure measures were not associated with resistance outcomes. These findings suggest that tetracycline treatment of feedlot cattle can be associated with modest increases in risk for recovery of resistant NTSEC, but the numerous treatments with an advanced macrolide (tulathromycin) were not associated with detectable increases in resistance in NTSEC. All cattle were exposed to in-feed treatments of tetracycline and this could limit the ability to identify the full impact of these exposures, but these exposures varied for enrolled cattle varied, providing an opportunity to evaluate a dose response. While AMD exposures were not associated with detectably increased risks for resistance to critically important AMDs, rare resistance outcomes and infrequent exposure to other important AMDs (e.g., cephalosporins) limited our ability to rigorously investigate questions regarding factors that can influence resistance to these important AMDs.

## Introduction

The development of antimicrobial resistance (AMR) is a complex multifactorial process driven by numerous and varied factors; however, exposure to antimicrobial drugs (AMDs) is clearly believed to play a large role in this development [1, 2]. Some scientists and public health officials are concerned that the use of AMDs in agricultural animals could be driving the development of resistant organisms that are then disseminated within human populations [3–6]. However, to truly understand and quantify this risk, we must first accurately characterize the causal associations between use of AMDs and AMR within animal production settings. This understanding will ideally allow development of reasonable and effective control measures [7]. Studies of AMR in populations of swine, poultry, and cattle have highlighted a number of factors that have been associated with variability in AMR. Specifically, such studies have described differences in exposures to AMDs, differences in management practices that might contribute to AMR, and differences in the detectable associations between resistance and exposures to AMDs [8–13]. Additionally, studies of temporal trends in the development of AMR have demonstrated that exposure to certain AMDs may only be associated with resistance in a transient fashion [7, 8]. Long-term surveillance studies are therefore needed to both monitor changes in resistance prevalence, and to better understand potential associations between exposure to AMDs and the development of AMR.

However, there are numerous challenges to studying relationships between AMD exposures and AMR [14–16]. Quantifying exposures to AMDs in a manner that provides the most relevant measure regarding selection pressures that could promote resistance is deceptively complex [17], as is representing antimicrobial susceptibility of bacterial isolates in a manner that is both relevant and also allows valid statistical analysis. Outcome measures of susceptibility testing can be presented in continuous (e.g., zone diameter) or categorical (e.g., breakpoint categories such as “susceptible”, “intermediate”, and “resistant”) representations, but the impact of choosing one method over another in relation to the impact on valid analysis of resistance ecology is unknown [18, 19]. Furthermore, analytic techniques for modeling these measures have inherent limitations and shortcomings. Giving full consideration to these challenges, the primary objectives of this study were to employ various analytical approaches to both estimate resistance prevalence and investigate AMD-AMR associations in non-type specific *Escherichia coli* (NTSEC) recovered from feces of individual feedlot cattle.

## Methods

### Overview

This study was a part of a large surveillance project conducted in western Canada. Details regarding the study population, sampling procedures, laboratory procedures, and interpretive criteria for antimicrobial susceptibility have been previously published [20]. Briefly, individual cattle were randomly enrolled and feces were obtained per rectum twice over the course of the study: during initial arrival processing and again later in the feeding period. Fecal samples were cultured to recover NTSEC, and the antimicrobial susceptibility of these isolates was evaluated using standardized panels of AMDs. Cattle were also sampled to recover isolates of *Mannheimia haemolytica*, with the goal of comparing resistance between a common pathogen and a common non-pathogen [13]. Exposures to AMDs were summarized from arrival until collection of the second sample for all enrolled cattle as well as for their pen-mates. Logistic regression was used to estimate resistance prevalence over time, and to evaluate associations between exposures to AMDs and the likelihood of detecting resistant isolates.

### Ethics Statement

Feedlots were recruited for participation by collaborators from a veterinary consulting company (Feedlot Health Management Services, Okotoks, Alberta, Canada; FHMS), and owners of the four participating feedlots gave explicit permission to work on the premises and to sample cattle that were enrolled in the study. All cattle handling and sampling procedures were approved prior to the initiation of the study by the Animal Care Committee of the University of Calgary (Protocol Number M07031). Specifically, in accordance with these approved protocols, cattle were restrained in approved animal handling facilities: trained personnel working under the supervision of licensed veterinarians collected fecal samples per rectum and long guarded swabs were used to sample deep in the nasopharynx.

### Study Population and Management

Details regarding the study population, sampling procedures, and laboratory procedures were published previously [20]. Briefly, study cattle were procured and managed at four commercial beef feedlots in south-central Alberta. Cattle were sourced from across Canada through the auction market system, and entered the feedlots at a range of weights (typically 225–400 kg), age classes, frame sizes and sexes. Cattle were processed at the time of arrival to feedlots to examine and treat ill cattle, and to administer standardized preventive and prophylactic treatments. Feedlots employed production practices typical for large feedlots located throughout western Canada and the U.S. Based upon these factors and historical patterns of illness in cattle of different types and sources, veterinary consultants (FHMS) classified arriving cattle according to perceived risk for developing bovine respiratory disease (BRD; very low risk to very high risk), and these classifications were used in assignment of prevention and treatment protocols. During arrival processing, all cattle received a hormonal implant, topical anthelmintic, and vaccines against selected pathogens (e.g., clostridial diseases, and cattle classified as having a very high risk of BRD received *M*. *haemolytica* anti-leukotoxin vaccine). Additionally, cattle classified as having high risk of developing BRD, or any that were exhibiting signs of systemic illness or fever also received metaphylactic or therapeutic AMDs during initial processing; lower risk cattle and cattle without clinical BRD were not treated with AMDs at arrival (S1 Table). Metaphylactic and therapeutic treatment protocols differed by risk status; cattle in higher-risk categories received drugs shown to have greater efficacy for prevention and treatment of respiratory disease [20–23]. Cattle were fed a diet that met or exceeded the National Research Council requirements for beef cattle [24] until reaching a body weight of 550–650 kg, at which time they were sent to slaughter, typically 120–250 days after arrival in the feedlot.

**Fig 1:**
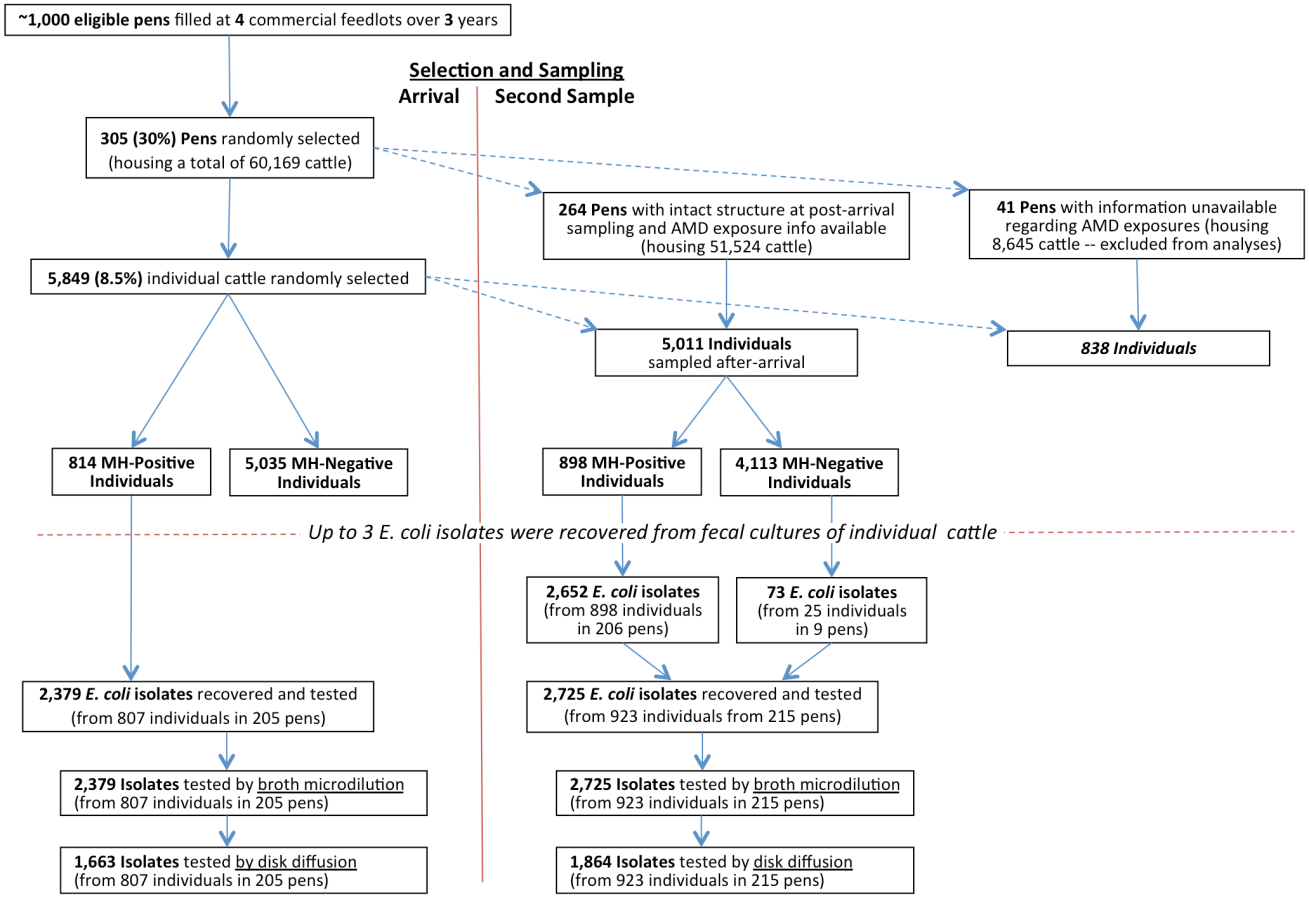
Selection of study pens, individuals and *E*. *coli* isolates at arrival and second sampling.

After initial processing, cattle were grouped in pens for housing through the feeding period. Most pen populations remained intact throughout the feeding period but a minority were subsequently split or merged with animals from other pens prior to harvest to facilitate marketing of similarly sized cattle at the time of harvest (Fig 1). The health of cattle was evaluated daily by trained feedlot personnel, and animals deemed to be sick were treated under the supervision of veterinarians from FHMS using standardized protocols (S1 Table). Additionally, all cattle received AMDs in feed as prophylaxis for liver abscesses. Cattle were enrolled from September 2007 to January 2010 using two-stage random sampling. As cattle arrived at the feedlots, 30% of all newly formed pens were randomly selected for inclusion, and approximately 10% of all cattle housed in selected pens were randomly enrolled (Fig 1).

### Sampling and Microbiology

Samples were collected as cattle arrived at the feedlot, and then at a second time point later in the feeding period when cattle were rehandled for routine production practices (e.g., placement of hormone implants). To facilitate analysis, the time of sampling relative to the arrival at feedlots was categorized (arrival or 0 days on feed [DOF], 33–75 DOF, 76–120 DOF, or >120DOF). Fecal samples were collected per rectum, and swab samples were collected from deep in the nasopharynx using 22 cm guarded swabs [13, 20]. Swabs were processed to recover *M*.*haemolytica* [25] and fecal samples were held until culture status for *M*. *haemolytica* was determined. As a standard protocol for this surveillance project, fecal samples from individual cattle were only processed to recover NTSEC isolates if *M*.*haemolytica* was cultured from that animal’s nasopharyngeal swab (Fig 1). This approach was used to conserve resources and to allow for comparison of resistance outcomes of both NTSEC and *M*.*haemolytica* obtained from the same animals [20]. We previously demonstrated that this protocol was not associated with systematic differences in susceptibility results of NTSEC isolates [20]. As such, the 90 NTSEC isolates recovered from a convenience sample of 25 *M*.*haemolytica*-negative cattle that were used in that evaluation were included in the study described herein (Fig 1).

One to 3 isolates of NTSEC were selected from each cultured sample and tested for antimicrobial susceptibility. Overall, resistance was assessed to 19 total drugs: all NTSEC isolates were tested for susceptibility to 15 drugs by broth microdilution (standard surveillance plate: Sensititre^®^ NARMS Gram Negative Plate Format, TREK Diagnostic Systems), and a convenience sample of isolates (at least one isolate from each sample) were tested for susceptibility to 11 drugs using disk diffusion (7 drugs included in the broth microdilution plate for method comparison purposes, plus 4 additional drugs of interest; S2 and S3 Tables). Susceptibility testing was conducted using protocols that adhered to standards delineated by the Clinical and Laboratory Standards Institute (CLSI), as previously described [18, 20, 26–28]. Because the majority of isolates were obtained from cattle that were culture-positive for *M*. *haemolytica*, isolates included in this study from the first sampling were not necessarily recovered from cattle that provided isolates obtained from at the second sampling. Breakpoints used to categorize susceptibility of isolates were obtained from published interpretive criteria [18, 20, 26–28] (S2 and S3 Tables).

### Antimicrobial Use Data

Data regarding exposure of individual cattle to AMDs were recorded at each feedlot throughout the study using chute-side computers and a customized information management system (*i*FHM*S*, Feedlot Health Management Services, Okotoks, Alberta). Exposures were recorded for cattle enrolled in the study as well as their pen-mates. These data included the unique animal identification, the AMD product used, dose, route of administration, and the date administered. Ionophore and coccidiostat exposures were not evaluated in this study. All study data were subsequently compiled in a computer spreadsheet and entries were verified.

Dosage information for each AMD was converted into an Animal defined Daily Dose (ADD), which represents the number of days that therapeutic concentrations are achieved in the target tissues of feedlot cattle upon administration of a single dose (S1 Table) [10, 11, 18, 20]. Drugs dosed at lower dosages were converted to partial ADDs relative to the approved labeled dosage that is recommended to achieve therapeutic concentrations for treatment of clinical infections (S1 Table). ADDs were then summed within animals by drug class and route of administration (i.e., parenteral exposures to beta lactams, macrolides, phenicols, quinolones, sulfonamides, and tetracyclines; in-feed exposures to macrolides and tetracyclines) for analysis purposes. In addition to evaluating the impact of AMD exposures in the enrolled cattle, we also evaluated impacts of exposures that may have occurred indirectly through treatment of the penmates of enrolled cattle. Therefore, aggregate (pen-level) exposures were summarized using treatment information for all cattle that were housed in pens containing study subjects. It was not possible to accurately document pen-level exposure information for the subset of pens that were split or merged with other pens, and animals housed in these pens were excluded in analyses investigating associations between resistance and AMD exposures.

The best method for summarizing these indirect (i.e., pen-level) AMD exposures has not been established; therefore, the group-level aggregate exposures were calculated in two ways: first, by dividing the summed pen-level exposures by the number of cattle housed in that pen; and second, in order to standardize in relation to the duration of the feeding period, through dividing the summed pen-level exposures by the sum of days that cattle had been housed in a pen prior to sampling. These exposure density estimates were assumed to provide representative measures of the ecological pressure that might be exerted on a pen of cattle beyond the exposure of individual study subjects to the AMDs. All exposures to AMDs between the 1st and 2nd sampling points were summarized for individuals by class of AMD, by route of administration (parenteral vs. in-feed), and by exposure context (exposures to individual study subjects vs. aggregate exposures for pens). To investigate whether AMDs given temporally close to sample collection elicited a fundamentally different AMR response in fecal NTSECs than AMDs given further back in time, we further stratified AMD exposures into three temporal periods. Period 1 represented AMDs given < 3 days prior to the second sampling, period 2 represented exposures 4 to 14 days prior to the second sampling, and period 3 represented AMDs given >14 days prior to the second sampling.

### Statistical Analysis

#### Antimicrobial Resistance Prevalence Estimates

**Fig 2:**
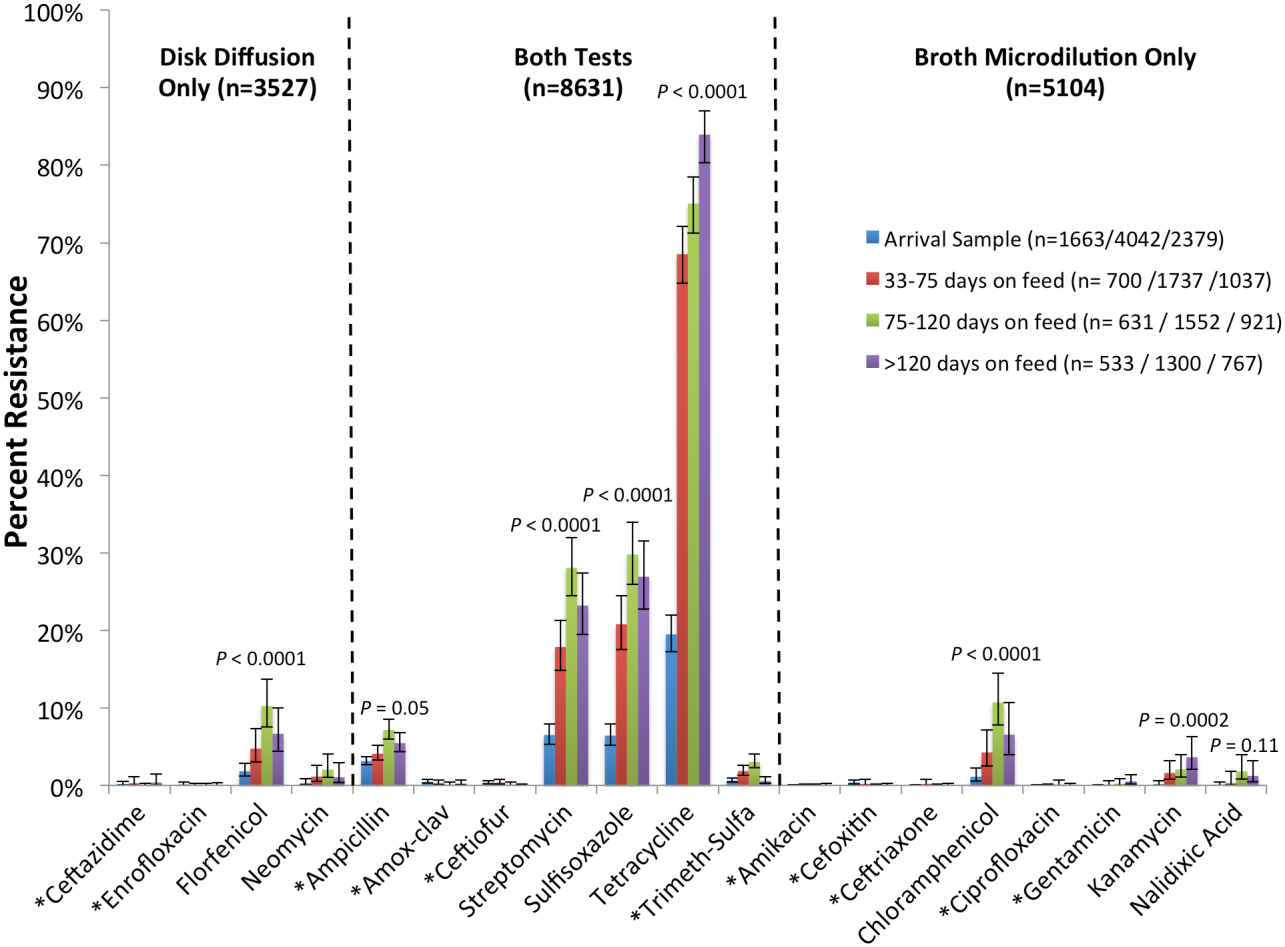
Prevalence of resistance in non-type-specific *E*. *coli* recovered from feedlot cattle, by sampling date. Marginal (adjusted) means estimates of the prevalence of resistance to various antimicrobial drugs among non-type specific *E*. *coli* isolates obtained from individual fecal samples at the first and second samplings. These estimates have been adjusted for isolate, individual, and pen effects. Due to a large variation in second sampling relative to days on feed, estimates have been categorized and presented at 33–75 days on feed, 75–120 days on feed, and >120 days on feed. Error bars represent 95% confidence intervals. Dashed lines differentiate which antimicrobial drugs were tested by one or both susceptibility testing methods. Number of isolates in legend indicate how many were tested by each susceptibility test (n = number tested by disk diffusion / number tested by both tests / number tested by broth microdilution). *P*-values relate to differences in adjusted prevalence among the 4 days-on-feed categories, and were not adjusted for multiple comparisons among AMDs. * = unadjusted prevalence with “plus four 95% confidence interval for a proportion".

To facilitate analyses, susceptibility outcomes for AMDs were dichotomized as resistant or non-resistant (the latter included both intermediate and susceptible classifications). It was not possible to estimate the prevalence of isolates resistant to amikacin, as the range of dilutions included on the commercial plate did not include the resistance breakpoint for that drug. Additionally, multivariable logistic regression models would not converge for 10 additional antimicrobials with low resistance prevalence (less than ~1.5%; Fig 2); therefore, unadjusted prevalence estimates and width-adjusted 95% confidence intervals (95%CI) for binomial proportions (adding 2 successes and 2 failures) are presented for these drugs [29]. For all other antimicrobials, the prevalence of resistance was estimated from marginal (adjusted) means (with 95%CI) using generalized estimating equations (GEE; PROC GENMOD, SAS^®^ version 9.4, SAS Institute Inc, Cary, NC) to control for lack of independence created by sampling and population structure. Compound symmetry (exchangeable) correlation structures were used to nest sets of isolates obtained from unique groups (pens) of cattle. In addition, for 7 drugs, some isolates were tested by both disk diffusion and broth microdilution (Fig 2, S2 and S3 Tables), and we controlled for these repeated measures using alternating logistic regression (ALR) with isolate ID specified as the subcluster [30]. For these GEE models, the time period of sampling during the feeding period (first sample obtained at arrival, second sample obtained at 33–75 DOF, second sample 76–120 DOF, or second sample >120 DOF) was used as the predictor variable of interest in order to evaluate longitudinal changes in resistance prevalence. Separate (parallel) models were developed to estimate adjusted resistance prevalence for the 8 AMDs for which models would solve (Fig 2).

#### Associations Between Resistance and Antimicrobial Exposures

Associations between AMD exposures in sampled cattle and resistance in fecal isolates were modeled using commercially available software (PROC GENMOD, SAS^®^ version 9.4, SAS Institute Inc, Cary, NC). Susceptibility to each drug (n = 19) that was tested was modeled separately using GEE regression modeling with a binomial distribution and a compound symmetry correlation structure to account for the lack of independence associated testing of multiple isolates from the same animal, and evaluating the isolates with two testing methods. It was not possible to investigate associations between exposure and resistance for 2 antimicrobials (enrofloxacin and amikacin); there was no resistance detected for enrofloxacin and the range of amikacin dilutions included on the commercial plate did not include the resistance breakpoint for that drug. As such, there was no dichotomy in the outcome for these drugs (i.e. no resistance was detected in the population leading to quasi-complete separation in logistic regression models). Thus associations between AMD exposures and AMR could only be investigated for 17 of the AMDs evaluated. Additionally, 3 of the resistance outcomes with sufficient resistance prevalence to allow modeling (streptomycin, sulfisoxazole, and tetracycline) were included on both the disk diffusion and broth microdilution susceptibility panels. Therefore, to model resistance outcomes for these drugs, ALR with GEE was used to account for the lack of independence associated with testing the same isolate using both susceptibility methods.

The primary exposure variables of interest in these regression models were the total ADDs of AMDs that enrolled cattle were exposed to directly and indirectly through their pen-mates (excluding pens that were split or merged prior to obtaining their second set of samples). To facilitate this modeling, AMDs were categorized into 6 drug classes with 2 routes of administration (parenteral and in-feed), and the 3 categories of exposure timing relative to sampling described above. Feedlot identification was included in all final models as a fixed effect. Antimicrobial drug exposures were initially screened individual for inclusion in multivariable model building using a liberal critical alpha for inclusion of 0.25. Backward selection was then used to develop final multivariable models with a critical alpha for retention of 0.05. Final models were assessed for confounding by adding previously eliminated variables back into the model one at a time and evaluating changes in the parameter estimates of all variables in the model. A change of ≥20% was considered evidence of confounding, and such variables were included in the final model. First order interactions of all main effects were then investigated using a critical alpha of 0.05. Any variables displaying characteristics of instability (e.g., extreme parameter estimates or confidence intervals) were removed from the modeling process. Odds ratios, 95%CI, and the associated *P*-values were reported from regression models.

#### Hierarchical Dependence

There was a complex hierarchical structure for the data collected for this study: multiple animals were enrolled per pen, multiple samples were collected per animal, multiple isolates were characterized from samples, and isolates were tested with two susceptibility testing methods. In addition to the potential effects on variance estimates, it was of interest for surveillance purposes to evaluate the ability to predict the resistance status of one isolate based upon the resistance status of isolates from within the same level of clustering. In order to characterize the relatedness (dependency or clustering) of susceptibility results within the 3 hierarchical levels of population organization listed above, null models were analyzed for each of the AMR outcomes using alternating logistic regression (PROC GENMOD, SAS v. 9.4, SAS Institute, Inc., Carey, NC; note multilevel mixed models would not solve for this data structure). Only a single subcluster could be analyzed using ALR in any given model, and thus outcome dependence in the different levels of clustering was assessed individually by using parallel analyses in which the different hierarchical clustering levels were coded as either repeated measures in GEE models or as subcluster terms in ALR. The likelihood of detecting the same resistance result from another isolate from within the same hierarchical level was determined by interpreting the odds ratio and 95%CI for the subcluster α parameter [30].

#### Correlation Among Resistance Outcomes

Multivariable logistic regression can only be used to model a single outcome in any given model, and therefore cannot account for the potential relatedness between multiple outcomes. Resistance outcomes for 1 drug can be associated with resistance to other drugs due to the potential for co-selection and transfer of multiple resistance genes on mobile genetic elements. Therefore, we used multivariate logistic regression to analyze the potential correlation between different combinations of resistance outcomes (MLwiN version 2.31, Center for Multilevel Modeling, University of Bristol). The statistically significant drug exposures that were identified in multivariable GEE modeling described above were controlled in these multivariate models, as were hierarchical data structures for pens, individual cattle, and isolates. Feedlot was included as a fixed effect. Multivariate models were used to investigate correlation between five outcomes—resistance to tetracycline, streptomycin, sulfisoxazole, ampicillin, and chloramphenicol; resistance to the 14 other drugs was too rare to support model convergence.

## Results

A total of 305 pens of cattle were selected for enrollment in this study at the time of arrival. Of these, 87% (264/305) housing a total of 51,424 cattle remained intact through the feeding period (Fig 1). The remaining 13% (41/305) of pens housed 8,648 cattle at the time of original placement, but were split or merged with cattle from other pens before marketing, as described above. Of the 60,072 cattle originally housed in enrolled pens, 8.5% (5,849/68,814) were enrolled in the study (Fig 1). Of these cattle sampled at arrival to feedlots, 13.9% (814/5849) of individuals housed in 205 pens were culture-positive for *M*. *haemolytica*, and their feces were cultured to recover NTSEC as described. Broth microdilution was used to evaluate susceptibility of all 2,379 NTSEC isolates recovered from arrival fecal samples, and disk diffusion was used to evaluate a subset of 69.9% (1,663/2,379) of these isolates (at least 1 isolate from each sample). The timing of collection of the second sample set varied across pens, ranging from 33–202 DOF (mean = 96 DOF, median = 80 DOF). For this second sample set, 17.9% (898/5,011) of cattle housed in 206 of the intact pens were culture-positive for *M*.*haemolytica*, as were 11.0% (92/838) of individuals that were housed in pens that were split or merged before marketing (Fig 1). A total of 2,725 NTSEC isolates were recovered from cattle housed in intact pens at the time the second sample set was collected, and all were tested with broth microdilution, while disk diffusion was used to evaluate a subset of 68.4% (1,864/2,725) of these isolates (at least 1 isolate from each sample). Seventy-three isolates recovered from 25 cattle that were culture-negative for *M*. *haemolytica*, as described above, also contributed to these totals.

### Antimicrobial Resistance

#### Unadjusted Resistance Prevalence

Despite using a slightly different panel of antimicrobial drugs, resistance phenotypes identified using broth microdilution were generally similar to those identified with disk diffusion, indicating that the more common resistance determinants were commonly represented by drugs included on both testing panels (Tables 1 and 2). Noting that there were differences in the number of drugs and specific makeup of testing panels, 79.8% (1988/2379) of NTSEC isolates in the first sample set that were tested with broth microdilution were pansusceptible and 98.6% (2346/2379) were resistant to ≤3 drugs, compared to 21.9% (597/2725) of the second sample that were pansusceptible and 92.7% (2346/2379) that were resistant to ≤3 drugs. In contrast, using disk diffusion testing, 76.5% (1272/1663) of isolates were pansusceptible on arrival samples and 98.1% (1632/1663) were resistant to ≤3 drugs. For the second sample set that were tested using disk diffusion, 26.3% (490/1864) were pan-susceptible and 92.9% (1731/1864) were resistant to ≤3 drugs. The most common phenotype for resistant isolates was single resistance to tetracycline, regardless of timing of sample collection, and tetracycline resistance was present in almost all of the most common phenotypes. The four most common multi-drug resistance patterns among all isolates were phenotype combinations that included resistance to streptomycin, sulfisoxazole, or tetracycline (Table 2).

**Table 1:** Number of antimicrobial drugs to which non-type specific *E*. *coli* isolates were resistant.

Test Method	Number of Resistant Drugs in Phenotype	Percent Resistance (n)^a^	
		First Sample Set	Second Sample Set
**Broth Microdilution** ^b^	Pansusceptible	79.8% (1898)	21.9% (597)
	1	11.2% (266)	41.8% (1138)
	2	4.0% (94)	18.8% (513)
	3	3.7% (88)	10.2% (279)
	4	0.9% (22)	5.0% (135)
	5	0.3% (7)	2.1% (57)
	6	0.0% (0)	0.1% (2)
	7	0.0% (0)	0.04% (1)
	8	0.1% (3)	0.04% (1)
	9	0.04% (1)	0.1% (2)
**Disk Diffusion** ^c^	Pansusceptible	76.5% (1272)	26.3% (490)
	1	12.9% (215)	37.1% (691)
	2	3.9% (65)	16.7% (311)
	3	4.8% (80)	12.8% (239)
	4	1.1% (19)	4.8% (90)
	5	0.5% (8)	1.9% (36)
	6	0.2% (3)	0.2% (4)
	7	0.0% (0)	0.1% (2)
	8	0.0% (0)	0.05% (1)
	9	0.1% (1)	0.0% (0)

^a^ Estimates were not adjusted for non-independence between isolates sampled from the same individual and between individuals from the same pen.

^b^ The susceptibility panel evaluated with broth microdilution evaluated 15 drugs (see S2 Table); n = 2379 for the first sample set, and n = 2725 for the second sample set.

^c^ The susceptibility panel evaluated with broth microdilution evaluated 11 drugs (see S3 Table); n = 1663 for the first sample set, and n = 1864 for the second sample set.

**Table 2:** Resistance patterns for non-type specific *E*. *coli* isolates recovered from the arrival and second sample sets.

Sample Set and Testing Method	Frequency	Percent of Isolates from Group^a^	Resistance Pattern^b^
**First Sample Set Using**			
**Broth Microdilution (n = 2379)**	1898	79.8%	Pansusceptible (i.e., no resistance detected)
	249	10.5%	Tet
	55	2.3%	Sulf-Strep-Tet
	47	2.0%	Sulf-Tet
	25	1.1%	Strep-Tet
	18	0.8%	Chlor-Sulf-Strep-Tet
	17	0.7%	Amp-Strep-Tet
	17	0.7%	Amp-Tet
	53	2.2%	Other Phenotypes^c^
**First Sample Set Using**			
**Disk Diffusion (n = 1663)**	1272	76.5%	Pansusceptible (i.e., no resistance detected)
	172	10.3%	Tet
	53	3.2%	Sulf-Strep-Tet
	23	1.4%	Sulf-Tet
	18	1.1%	Strep-Tet
	17	1.0%	Amp
	15	0.9%	Amp-Strep-Tet
	15	0.9%	Flor-Sulf-Strep-Tet
	12	0.7%	Amp-Tet
	9	0.5%	Sulf
	57	3.4%	Other Phenotypes
**Second Sample Set Using**			
**Broth Microdilution (n = 2725)**	597	21.9%	Pansusceptible (i.e., no resistance detected)
	1130	41.5%	Tet
	255	9.4%	Sulf-Tet
	183	6.7%	Strep-Tet
	178	6.5%	Sulf-Strep-Tet
	83	3.1%	Chlor-Sulf-Strep-Tet
	55	2.0%	Amp-Tet
	34	1.3%	Amp-Strep-Tet
	29	1.1%	Kan-Sulf-Strep-Tet
	23	0.8%	Chlor-Sulf-Tet
	19	0.7%	Chlor-Sulf-Strep-TMS-Tet
	15	0.6%	Amp-Sulf-Tet
	14	0.5%	Nal-Tet
	13	0.5%	Amp-Chlor-Sulf-Strep-Tet
	13	0.5%	Chlor-Sulf-Strep
	84	3.1%	Other Phenotypes
**Second Sample Set Using**			
**Disk Diffusion (n = 1864)**	491	26.3%	Pansusceptible (i.e., no resistance detected)
	675	36.2%	Tet
	164	8.8%	Sulf-Strep-Tet
	145	7.8%	Sulf-Tet
	112	6.0%	Strep-Tet
	66	3.5%	Sulf-Flor-Strep-Tet
	37	2.0%	Amp-Tet
	30	1.6%	Amp-Strep-Tet
	16	0.9%	Sulf-Flor-Tet
	14	0.8%	Sulf-Flor-Strep-TMS-Tet
	10	0.5%	Sulf-Neo-Strep-Tet
	9	0.5%	TMS-Tet
	95	0.4%	Other

^a^ Estimates were not adjusted for non-independence between isolates sampled from the same individual and between individuals from the same pen.

^b^ Amp = ampicillin; Chlor = chloramphenicol; Flor = florfenicol; Kan = kanamycin; Nal = naladixic acid; Neo = neomycin; Strep = streptomycin; Sulf = sulfamethoxazole; Tet = tetracycline; TMS = trimethoprim-sulfamethoxazole.

^c^ Other phenotypes were those that individually represented <0.5% of isolates.

#### Adjusted Resistance Prevalence

For drugs with relatively low resistance prevalence (ampicillin, amoxicillin-clavulanate, cefoxitin, ceftazidime, ceftiofur, ceftriaxone, ciprofloxacin, gentamicin, and trimethoprim-sulfamethoxazole), it was not possible to estimate resistance prevalences while adjusting for correlation related to the hierarchical population and sampling structure, for repeated testing of the same isolates with different susceptibility tests, and for feedlot-level effects (Fig 2). In other models that controlled for these effects, the most common resistances detected in the first sample set among the remaining 8 drugs included streptomycin (6.5%; 95%CI 5.3–8.0), sulfisoxazole (6.4%; 95%CI 5.2–7.9), and tetracycline (19.5%; 95%CI 17.3–21.9; Fig 2). These 3 drugs remained the most commonly detected resistance phenotypes throughout the feeding period, and their prevalence later in the feeding period increased significantly compared to the first sampling (22.8, 95%CI: 20.8–25; 25.6, 95%CI: 23.4–27.9; and 75.2, 95%CI: 73.1–77.3, respectively; Table 3). All other AMDs tested had resistance prevalence of <15% at both sampling points (Fig 2) but resistance for several of these drugs still increased significantly from first to second sampling (florfenicol, neomycin, chloramphenicol and kanamycin). None of the tested drugs exhibited a significant decrease in resistance prevalence between first and second sample sets (Fig 2).

**Table 3:** Exposures to antimicrobial drugs in individual cattle from which NTSEC were recovered for this study (n = 923).

Antimicrobial Drug Exposure		Days Prior to Second Sample Collection											
Route of Administration	Class	0 to 3			4 to 14			>14			Total		
		*n* ^*a*^	*%* ^*b*^	*ADDs* ^c^	*n*	*%*	*ADDs*	*n*	*%*	*ADDs*	*n*	*%*	*ADD*
**Parenteral**													
	Beta lactam	0	0.0%	0	2	0.2%	6	15	1.6%	35	17	1.8%	41
	Macrolide	0	0.0%	0	0	0.0%	0	186	20.2%	504	186	20.2%	504
	Phenicol	0	0.0%	0	1	0.1%	3	13	1.4%	39	14	1.5%	42
	Quinolone	0	0.0%	0	0	0.0%	0	4	0.4%	12	4	0.4%	12
	Sulfonamide	0	0.0%	0	0	0.0%	0	3	0.3%	9	3	0.3%	9
	Tetracycline	0	0.0%	0	0	0.0%	0	258	28.0%	751	258	28.0%	751
**In-Feed**													
	Macrolide	30	3.3%	1.1	21	2.3%	2	91	9.9%	9.8	102	11.1%	12.8
	Tetracycline	894	96.9%	146.8	910	98.6%	553	923	100.0%	7751.5	923	100.0%	8451.5

NTSEC = non-type-specific *E*. *coli*

^a^ Number of enrolled cattle that received AMDs among those from which NTSEC were recovered and used in these analyses

^b^ Percent of exposed cattle relative to the number of enrolled cattle from which NTSEC were recovered and used in these analyses (n = 923)

^c^ Sum of ADDs administered to enrolled cattle. ADD = animal defined dose, or the amount of drug needed to achieve therapeutic concentrations in target tissues for one day.

### Antimicrobial Drug Use

At the time of collection of the second sample set, all cattle had been exposed to varying doses of AMDs prior to collection. All had received low doses of AMDs daily in feed as a preventative for liver abscesses, a common health problem in feedlot cattle (S1 Table and Table 3). A subset of these cattle, 44.6% (412/923) of the enrolled individuals, were treated parenterally with AMDs, most commonly for treatment and prevention of respiratory disease (min = 1 ADD for treated cattle, mean = 3.3 ADDs, median = 3 ADDs, max = 13 ADDs). Table 4 shows the average exposure for all cattle housed in pens where AMDs were used to treat resident cattle. Overall, prior to collection of the second sample set, all cattle housed in enrolled pens were exposed to an average of 11.3 ADDs of AMDs of all classes through parenteral and in-feed exposures. Enrolled cattle received an average sum of 9 ADDs of tetracycline in feed prior to collection of the second sample set (range 2.0–37.4 ADDs; Table 3). Macrolides and tetracycline were the drugs most commonly administered parenterally, and the majority of these parenteral exposures occurred >14 days prior to sampling. Parenteral treatment with beta lactam, phenicol, quinolone, and sulfonamide drugs occurred in <2% of the enrolled cattle (Table 3).

**Table 4:** Pen-level exposures to antimicrobial drugs for groups which housed cattle that were used (n = 215 pens).

Antimicrobial Drug Exposures		Pens Exposed		Averages for Cattle Housed in Exposed Pens^a^	
Route of Administration	Class	n^a^	%^b^	Average ADD per Animal^c^	Average ADD Per Animal-Day^d^
**Parenteral**					
	Beta lactam	153	71.2%	0.0573	0.0006
	Macrolide	131	60.9%	0.7199	0.0075
	Phenicol	65	30.2%	0.0713	0.0007
	Quinolone	76	35.3%	0.0421	0.0004
	Sulfonamide	117	54.4%	0.036	0.0004
	Tetracycline	153	71.2%	1.1048	0.0116
**In-Feed**					
	Macrolide	34	15.8%	0.0812	0.0009
	Tetracycline	215	100.0%	9.1825	0.0962

^a^ Number of pens where enrolled cattle (n = 923 from which NTSEC were recovered and used in analyses) received antimicrobial drugs.

^b^ Percent of pens relative to the total number that housed cattle from which NTSEC were recovered and used in analyses (n = 215).

^c^ Pen-level average of the Animal Defined Doses (ADDs) of antimicrobial drugs administered to all cattle in housed in pens (total cattle n = 42,729 from n = 215 pens).

^d^ Pen-level average of the ADDs received per animal, per day prior to the date when the second sample set was collected for a pen. Overall, the average time from arrival until sample collection was 95.5 days.

Cattle in the study population were most commonly exposed to tetracyclines and macrolides during the study period (Tables 3 and 4). The majority of pens housed at least one animal that was treated with parenteral AMDs before second sampling. However, in most of pens where at least one animal was treated parenterally, only a very small number of cattle were treated. Therefore, the average indirect exposure for an individual in such pens was very small (Table 4), especially when compared to the amount of direct exposure that cattle received if they themselves were treated parenterally. In addition, almost all exposures to parenteral AMDs occurred shortly after cattle arrived in the feedlot, and therefore were not temporally close to second sampling (i.e., treatments typically preceded second sampling by much >14 days). Exposures to in-feed tetracycline occurred in all cattle throughout the feeding period.

### Associations between Antimicrobial Use and Resistance in Isolates from Second Samples

In a subset of isolates recovered from the second sample set, there were no resistant isolates identified for 1 of the 18 AMDs evaluated in this investigation (enrofloxacin), and it was not possible to estimate the prevalence of isolates resistant to amikacin as the range of dilutions included on the commercial plate did not include the resistance breakpoint. Additionally, the resistance prevalence was <0.3% for 7 additional AMDs; there were <10 resistant isolates identified for ciprofloxacin (n = 1), ceftriaxone (n = 2), cefoxitin (n = 2), ceftazidime (n = 4), ceftiofur (n = 7), gentamicin (n = 7), and amoxicillin-clavulanate (n = 9). Because of rarity of these outcomes, multivariable logistic regression models could not be used to investigate these associations. We attempted to investigate whether the timing of AMD exposures (i.e., temporal distance between exposure and sampling) affected the likelihood of treatments being associated with resistance. However, almost all parenteral exposures occurred >14 days prior to sampling, and all of the cattle were exposed to AMDs in-feed throughout the feeding period. As such, it was not possible to investigate whether differences in the elapsed time from treatment affected the strength of these associations. Additionally, for the 6 classes of parenteral drug exposure and the two classes of in-feed exposure that were quantified, not all exposures to AMDs occurred with sufficient frequency to permit regression models to solve reliably.

For resistance outcomes regarding the 10 drugs that could be modeled, all associations could be evaluated for parenteral exposures to tetracyclines (exposures in study subjects and pen-mates) and macrolides (exposures in study subjects) and in-feed exposures to tetracyclines (exposures in study subjects and pen-mates). A limited number of associations could be investigated in regression models for the other exposures (n = 8 models solved for parenteral beta-lactam exposures in study subjects and n = 9 for exposures in pen-mates; n = 7 models solved for parenteral exposures to phenicols in study subjects; n = 7 models solved for parenteral sulfonamide exposures in pen-mates and n = 5 models solved regarding exposures in study subjects; n = 4 models solved for parenteral quinolone exposures in pen-mate and n = 3 models solved for exposures in study subjects; n = 8 models solved for in-feed macrolide exposures to both study subjects and pen-mates).

For these 10 resistance outcomes and the limited number of exposures that could be modeled, only exposures to tetracycline, streptomycin, and trimethoprim-sulfamethoxazole were significantly associated with AMR outcomes (Table 5). Every additional 3 ADD of tetracycline administered parenterally or 7 ADD of in-feed exposure to an enrolled animal were associated with increased odds of recovering tetracycline-resistant isolates when compared to unexposed cattle (respectively: OR = 1.3, 95%CI = 1.01–1.73, *P* = 0.04; and OR = 1.2, 95%CI = 1.03–1.35, *P* = 0.01, respectively). Similarly, direct exposure to parenteral tetracycline increased the likelihood that a steer’s fecal sample isolate would exhibit phenotypic resistance to trimethoprim-sulfamethoxazole (OR = 2.6, 95%CI = 1.72–3.89, *P* = 0.001; Table 5). Interestingly, the odds of recovering streptomycin-resistant NTSEC were about 3 times lower (OR = 0.3; 95%CI = 0.08–1.25; *P* = 0.01) in cattle that had received parenterally-administered beta-lactam drugs (when controlling for the use of parenteral sulfonamides as a confounding variable). Indirect (i.e. pen-level) exposure to AMDs were not associated with resistance in NTSEC isolates recovered from study animals.

**Table 5:** Final multivariable logistic models of associations between antimicrobial drugs and antimicrobial resistance.

Resistance Outcome Variable	Exposure Variable	Odds Ratio^a^ (95%CI)	*P*-value
Tetracycline	Individual Parenteral Tetracycline	1.32 (1.01–1.73)	0.04
	Individual In Feed Tetracycline	1.18 (1.03–1.35)	0.01
Streptomycin^b^	Individual Parenteral Beta lactam	0.32 (0.08–1.25)	0.01
Trimethoprim-Sulfamethoxazole	Individual Parenteral Tetracycline	2.59 (1.72–3.89)	0.001

Population-averaged odds ratios and 95% confidence intervals (95%CI) are presented relative to an exposure of 3 ADD (animal defined daily dose) treatment of parenteral antimicrobials or a 7 ADD exposure to in-feed antimicrobials.

^a^ Results of logistic regression modeling using generalized estimating equations (GEE) and alternating logistic regression for the outcomes of tetracycline and streptomycin resistance; controls for 1 level of clustering (individuals) with 1 subcluster (isolates). The outcome of trimethoprim-sulfamethoxazole resistance was analyzed using regular GEE regression models controlling for 1 level of clustering (isolates). Feedlot ID was controlled as a fixed effect in all models.

^b^ Model included the variable for individual exposures to parenteral sulfonamides as a confounding variable (*P* = 0.19).

### Hierarchical Dependence

The correlation between susceptibility outcomes for isolates recovered within the same pen, the same individual, or for repeated testing of the same isolates could only be evaluated for florfenicol, streptomycin, sulfisoxazole, tetracycline, and chloramphenicol; resistance prevalences for all other tested AMDs were too low to support model convergence. Generally, the resistance status of an NTSEC isolate had little ability to predict the resistance status of an NTSEC isolate recovered from another animal housed in the same pen (Table 6). However, multiple NTSEC isolates recovered from the same individual were more likely to have the same resistance status, and susceptibility results for the same NTSEC isolate were very highly correlated when tested with both broth microdilution and disk diffusion.

**Table 6:** Dependence at different levels of clustering for NTSEC isolates in the second sample set as estimated using alternating logistic regression^a^.

Antimicrobial Drug Resistance^b^	n^c^	Resistance Prevalence^d^	(95%CI)	Dependence in Resistance Results^a^					
				Pen^e^		Individual^e^		Isolate^e^	
				*PWOR*	*(95%CI)*	*PWOR*	*(95%CI)*	*PWOR*	*(95%CI)*
Amoxicillin-Clavulanate	8631	0.3%	(0.1–0.5%)	9.1	(4.5–18.4)	NE^f^		NE	
Ampicillin	8631	4.7%	(4–5.4%)	2.9	(1.8–4.6)	NE		NE	
Cefoxitin	5104^g^	0.2%	(0.1–0.4%)	10.2	(4.8–21.8)	NE		—^g^	
Ceftiofur	8631	0.3%	(0.1–0.5%)	5.6	(1.4–22.7)	NE		NE	
Chloramphenicol	5104^g^	4.4%	(2.4–7.8%)	3.6	(3.2–4.1)	NE		—^g^	
Florfenicol	3527^h^	5.0%	(3.1–8.1%)	3.0	(2.7–3.4)	NE		—^h^	
Neomycin	3527^h^	1.0%	(0.5–1.8%)	2.4	(1.2–4.8)	NE		—^h^	
Streptomycin	8631	17.5%	(14.3–21.4%)	1.9	(1.6–2.2)	10.9	(9.3–12.7)	65.2	(61.1–69.7)
Sulfisoxazole	8631	19.5%	(15.6–24.5%)	1.7	(1.3–2.1)	11.7	(10–13.8)	243.7	(141.1–421.1)
Tetracycline	8631	NE		2.0	(1.8–2.1)	9.8	(8–12)	138.3	(93.7–204.3)

^a^ Pairwise odds ratios (PWOR) and 95% confidence intervals (95%CI) of null models are presented for each hierarchical level.

^b^ The prevalence of resistance to the 9 other antimicrobial drugs was too low to allow estimation of PWOR for clustering.

^c^ Numbers of susceptibility tests performed using either broth microdilution or disk diffusion. Some isolates were tested using both methods.

^d^ The adjusted prevalence is averaged across all days-on-feed categories (arrival, 33–75, 76–120, and >120 DOF) and controls for the population hierarchy at different indicated levels within a feedlot.

^e^ PWOR and 95%CI regarding the likelihood of obtaining the same results (resistant or non-resistant) for two tests randomly selected from the same pen, from the same individual animal, or for repeated testing of the same isolate.

^f^ Not estimable because of low resistance prevalence.

^g^ Susceptibility was only tested by disk diffusion.

^h^ Susceptibility was only tested by broth microdilution.

NE = not estimable

### Correlation Among Resistance Outcomes

The multivariate model revealed varying degrees of correlation between resistance to tetracycline, streptomycin, sulfisoxazole, ampicillin, and chloramphenicol (Table 7). The relationship between streptomycin resistance and sulfisoxazole resistance was stronger than between any other combination of antimicrobial drugs in this model (ρ = 0.50; SE = 0.02). Not surprisingly, resistance outcomes with the strongest correlation were also identifiable in most common resistance phenotypes identified among NTSEC isolates (Table 2).

**Table 7:** Multivariate correlation between resistance outcomes. Pairwise correlation between 2 antimicrobial resistance outcomes obtained from a multivariate regression model including resistances to tetracycline, streptomycin, sulfisoxazole, ampicillin, and chloramphenicol.

Antimicrobial Drug Resistance Combination		Correlation	Standard Error	*P*-value
Streptomycin	Sulfisoxazole	0.50	0.02	<0.0001
Tetracycline	Sulfisoxazole	0.34	0.03	<0.0001
Sulfisoxazole	Chloramphenicol	0.33	0.03	<0.0001
Tetracycline	Streptomycin	0.33	0.03	<0.0001
Streptomycin	Chloramphenicol	0.27	0.03	<0.0001
Streptomycin	Ampicillin	0.16	0.03	<0.0001
Tetracycline	Chloramphenicol	0.12	0.03	0.0001
Tetracycline	Ampicillin	0.09	0.03	0.01
Ampicillin	Chloramphenicol	0.06	0.03	0.06
Sulfisoxazole	Ampicillin	0.05	0.03	0.15

## Discussion

Results of this study suggest that exposures to AMDs in feedlot cattle from the time of arrival to the time that the second set of samples were collected were not strong determinants of resistance when measured at this second sampling point. The few significant associations identified between antimicrobial exposures and resistances were relatively weak (i.e. odds ratios were close to 1) and did not relate to AMDs considered to be critically important to healthcare of people. However, it is important to note that low resistance prevalences for several drugs in combination with infrequent exposures to some classes of antimicrobial drugs limited the ability to explore all possible exposure-resistance relationships. Prevalence of resistance was negligible for all antimicrobials tested that are considered very important to critically important in human medicine (e.g., amoxicillin-clavulanate, 2^nd^ and 3^rd^ generation cephalosporins, fluoroquinolones, aminoglycosides). However, relatively high rates of resistance were observed for tetracycline in the second sample set, along with moderate resistance prevalence for streptomycin and sulfonamides. Similarly high prevalences of tetracycline resistance have been noted in comparable populations, especially those where tetracyclines are commonly administered [31]. However, other studies have found temporal trends for increasing resistance to tetracycline during the feeding period in feedlot cattle even in the absence of exposure to antimicrobials [10].

Confounding factors that were unmeasured in this study such as environmental, dietary, and other management pressures could have contributed to these relationships [7, 32, 33]. The relationship between tetracycline use and resistance may lack practical significance in relation to resistance among pathogenic bacteria since tetracyclines continue to be efficacious in feedlot populations for prevention and treatment of bovine respiratory disease complex, prevention of disease caused by *Histophilus somni*, and in control of liver abscesses [22, 23, 34]. We have previously shown that NTSEC are much more likely to be resistant than *M*. *haemolytica* recovered from the same cattle [20]. In fact, among *M*. *haemolytica* isolates obtained from these cattle at the time that fecal samples were obtained for this study, 87.8% were pan-susceptible, and only 4.4% were resistant to tetracycline, 4.2% to streptomycin, and 0.4% to sulfonamides [13]. While in-feed exposures in the whole population to tetracycline provide an opportunity to observe the magnitude of changes that might relate to these treatments, it is also possible that the selection pressures created by in-feed treatment of all animals may affect the impact that other AMD exposures might have had in the absence of selection pressure from continuous exposure to low-dose in-feed AMDs. Additionally, exposure of an entire population to the influence can mask our ability to identify relative differences within that population. However, while all cattle were exposed to tetracycline in their feed, these exposures varied considerably and thus it provided an opportunity of evaluating the dose-response effects.

This work represents one of the largest studies performed to date regarding antimicrobial resistance (AMR) in confinement-reared beef cattle. At the same time, this study represents one context regarding beef production and AMD exposures, and further research is needed to investigate the impacts of AMD exposures in other contexts. One of the limitations of previous observational studies examining AMR in food animals has been the ability to obtain detailed information about antimicrobial drug (AMD) use in individual animals [10, 11]. Our group and others have attempted to investigate associations between AMD use and AMR in feedlot cattle in previous field studies, but have been limited to examining drug exposures at the group level. Thus, having fecal samples obtained from individual animals, and also having the detailed AMD treatment information for these same individuals as well as all cattle housed in the same pens, provided a very unique and valuable opportunity to investigate these potential associations. Using this observational study design had obvious practical relevance as it was conducted in typical production settings. However, despite detailed prior planning and the huge effort involved in conducting this study, rare resistance outcomes and rare AMD exposures for some classes of drugs thwarted our ability to provide greater insight into important questions about critically important AMDs.

The administration of an AMD theoretically alters a bacterial population by eliminating the susceptible organisms and thus increasing the proportion of those organisms resistant to the antimicrobial among the remaining microbial population. A protective association was identified in this population between exposure to parenteral beta lactams and resistance to streptomycin. A strictly numerical interpretation of this relationship may be that the usage of parenteral beta lactams is associated with an increase in susceptibility to streptomycin. Theoretically, a more appropriate causal interpretation of this complex relationship may be that the proportion of bacteria susceptible/resistant to streptomycin was indirectly modified by these exposures. As was noted in the correlation between outcomes in the multivariate model, certain resistances are biologically linked and may persist in bacterial populations when selective pressures are applied due to their relationship with other resistances rather than strictly due to exposure to antimicrobial drugs. Not all potential contributors to AMR were included in this study, nor would it be possible to measure them all. It is possible that investigation of linked genetic elements through genomics approaches may help to provide a better understanding of these issues.

The association identified in this study between parenteral tetracycline exposures and trimethoprim-sulfamethoxazole resistance was identified when ignoring the individual level of clustering, and modeling only the dependence at the isolate level as the more complex model would not mathematically converge; this was likely due to the extremely low prevalence of resistance to trimethoprim-sulfamethoxazole in this population. Since this dataset includes relatively few isolates that are resistant to this drug, it is questionable that the estimate is highly accurate relative to the true association. It would be beneficial to see if this association holds true in a population with a higher prevalence of trimethoprim-sulfamethoxazole. Despite intentions to gather a robust dataset to address important questions about associations between antimicrobial resistance and exposure using state-of-the-art statistical analyses, model instability attributable to sparse outcome and exposure strata greatly limited our ability to accomplish this goal. Unfortunately, many of these rare resistance outcomes are of great concern from a public health perspective, and this limitation in our ability to investigate factors that might promote or protect against resistance propagation in normal production settings is an issue that needs further consideration and discussion.

Investigation of potential associations between resistance outcomes and AMD exposures or other explanatory factors is heavily reliant upon the use of advanced multivariable regression modeling, and we have shown that model selection can have a marked impact on the results [35]. Using logistic regression to analyze AMR data is heavily dependent on interpretive criteria (cut off values) used to classify bacteria as resistant or susceptible, and thus models may change if more liberal or conservative criteria are used. So-called “epidemiological” cutoffs are often much lower than cutoffs that are standardized for use regarding clinical treatment, so a regression technique capable of detecting a more subtle change in resistance might be preferred [36, 37]. With this capability, changing trends in resistance prevalence might be detected earlier. However, using non-standardized cutoff values is problematic, as results from one testing methodology cannot be easily extrapolated to other testing methodologies (e.g., results from broth microdilution vs. those from disk diffusion). Great efforts are taken to standardize methodologies to clinical outcomes, which allows standardization of quality control procedures and provides the greatest opportunity for assuring that similar results will be obtained from different laboratories and from different testing methodologies [18, 27]. Regression of a continuous outcome (e.g., zone diameter) rather than a categorized one (e.g., dichotomized interpretive criteria or MIC) would theoretically provide more detailed information [18, 38]. Unfortunately, in our preliminary modeling efforts the assumptions of linear regression could not be met for all drugs when using zone diameters obtained from disk diffusion testing as the model outcome. The disk diffusion zone diameters from this population of NTSEC had distributions that ranged from closely normal (e.g., enrofloxacin) to bimodal (e.g., sulfisoxazole), or even lacked an identifiable distribution (e.g., tetracycline). Even using transformation, the distributions of zone diameters could not be reliably or uniformly made to approximate Gaussian distributions. Additionally, the zone diameter data were not interval in nature, as a 1 mm difference in zone diameter at the top of the scale (35 mm vs. 36 mm) and a 1 mm difference at the bottom of the scale (7 mm vs. 8 mm) are not equivalent in relation to the differences in drug concentrations. Thus, while zone diameter measurements are continuous, they are not interval data, which are both assumptions of linear regression. Finally, a nonparametric rank analysis provided conservative levels of significance as well as a reliable estimate indicating the direction of association. However, the parameter estimates derived from analysis of rank-transformed data are not interpretable in a biologically relevant manner as the ranks are not interval in nature either (i.e., there is not a uniform difference in susceptibility associated with a one unit increase in rank). Thus, using methods that allow regression analysis of data that is not dichotomous may have theoretical advantages when investigating factors associated with susceptibility, but we were not able to identify other methods that could meet statistical assumptions and allow interpretations in a practically relevant manner.

Susceptibility testing in this study included 2 methods that are often recommended for surveillance programs. The results of both susceptibility tests were combined and reported here to facilitate the description of resistance to a wider range of antimicrobials and to increase the power in the analysis for the AMDs that had coverage in both panels. The results of both methods were dichotomized based on standardized interpretive criteria and could be easily incorporated into the same logistic models. Essentially, for the AMDs shared between the panels of these susceptibility tests, the dual testing of the same isolate provided a repeated measure which was controlled as a subcluster in ALR, or a lower hierarchical level in the multivariate model. Alternating logistic regression gives a more accurate estimate of the correlation/error structure than standard GEE models [30]. Evaluation of the hierarchical dependence at this level indicated that the status of resistance for the same isolate tested by one test is highly predictive of the status of resistance detected by the other test. Given the substantially higher costs per test for broth microdilution compared to disk diffusion, this similarity in test results is a critically important finding that corroborates findings of a previous study [18].

The lack of independence between isolates from the same individuals and individuals from the same pen was expected to account for some of the variability in the associations investigated in this study [38]. Nested effects for individuals in this analysis allowed for adjustment to account for this lack of independence between individuals. The authors also expected the baseline resistance status of each isolate as well as the extent of the association with exposures to AMDs to vary, so analysis with random intercepts and random slopes would be intuitively appealing. Additional variability due to the sampled individuals would have been accounted for with this methodology and models would have produced estimates theoretically closer to the true estimates. However, the random coefficient models could not be used with this particular data set because the models would not converge to produce estimates of association. Similar future analytical efforts should investigate this technique in other datasets to further expand our understanding of different sources of variability in antimicrobial resistance.

To reliably evaluate the association between AMR and exposure to all AMDs administered to an individual as well as to other cattle housed with the sampled individual, pens that were mixed or split during the feeding period were excluded from analyses. These management practices were applied to pens selected from all those enrolled in the study without regard for criteria related to antimicrobial exposures or illness, and no systematic demographic or exposure differences were identified between pens that were included or excluded from the analysis. The 4 large feedlots included in this study were purposefully selected because of their use of a computerized data system designed to allow recording of highly detailed antimicrobial exposure data (*i*FHM*S*). The use of this comprehensive data collection system may also have been a marker of other progressive management practices, and thus these feedlots may not be entirely representative of all feedlots in North America. However, field-based research regarding the effects of AMU in feedlot cattle necessarily requires accurate exposure data. Thus, the study population, sampling and laboratory methods and analysis procedures used in this study should be relevant to other studies of antimicrobial resistance in feedlot cattle in North America.

## Supporting Information

S1 Table Antimicrobial drugs used in the study population and the ADD assigned to a single treatment. (DOCX)

S2 Table Interpretive criteria for *E*. *coli* using broth microdilution susceptibility testing reported as minimum inhibitory concentrations (μg/ml). (DOCX)

S3 Table Interpretive criteria for *E*. *coli* using disk diffusion susceptibility testing reported as inhibition zone diameters (mm). (DOCX)
