# Supplementary material for: Antimicrobial Resistance in Escherichia coli Recovered from Feedlot Cattle and Associations with Antimicrobial Use
Source: PLoS One. 2015 Dec 3;10(12):e0143995. doi: 10.1371/journal.pone.0143995 (PMC4669080; doi:10.1371/journal.pone.0143995)
Supplement: S1 Table — (DOCX) [file pone.0143995.s001.docx]

**Table S1. Antimicrobial drugs used in the study population and the ADD assigned to a single treatment**

| **Antimicrobial Drug and Dosage** | **Primary Reason for Use** | **Class** | **ADD** |
| --- | --- | --- | --- |
|  |  |  |  |
| **Parenteral** |  |  |  |
| Ceftiofur sodium 1 mg/kg BW | BRD Treatment | Beta lactam | 1 |
| Ceftiofur crystalline free acid 6.6 mg/kg BW | BRD Treatment | Beta lactam | 3 |
| Ceftiofur hydrochloride 1.1 mg/kg BW | BRD Treatment | Beta lactam | 1 |
| Enrofloxacin 7.7 mg/kg BW | BRD Treatment | Quinolone | 3 |
| Florfenicol 40 mg/kg BW | BRD Treatment | Phenicol | 3 |
| Florfenicol 40 mg/kg BW and Flunixin meglumine 2.2 mg/kg BW | BRD Treatment | Phenicol | 3 |
| Oxytetracycline |  |  |  |
| 10 mg/kg BW | BRD Prevention & Treatment | Tetracycline | 1 |
| 20 mg/kg BW | BRD Prevention & Treatment | Tetracycline | 2 |
| 30 mg/kg BW | BRD Prevention & Treatment | Tetracycline | 3 |
| Tilmicosin 10 mg/kg BW | BRD Prevention & Treatment | Macrolide | 3 |
| Trimethoprim and sulfadoxine 16 mg/kg BW | BRD Treatment | Sulfonamide | 1 |
| Tulathromycin 2.5 mg/kg BW | BRD Prevention & Treatment | Macrolide | 3 |
| Tylosin tartrate 29 mg | Implant Site Abscess Prevention | Macrolide | 1/275 |
|  |  |  |  |
| **In-Feed** |  |  |  |
| Chlortetracycline @ |  |  |  |
| 35 mg/kg diet dry matter | Liver Abscess Prevention | Tetracycline | 1/18 ^a^ |
| 1 g/head/day | *Histophilus somni* Prevention & Treatment | Tetracycline | 1/6 |
| 3 g/head/day | *Histophilus somni* Prevention & Treatment | Tetracycline | 1/2 |
| 6 g/head/day | *Histophilus somni* Prevention & Treatment | Tetracycline | 1 |
| Tylosin phosphate @ 11 mg/kg diet dry matter | Liver Abscess Prevention | Macrolide | 1/80 ^a^ |
|  |  |  |  |

ADD = Animal Defined Daily Dose; BW = body weight; BRD = Bovine Respiratory Disease

^a^ Assuming 9 kg of dry matter intake per individual animal per day.
