# Supplementary material for: Antimicrobial Resistance in Escherichia coli Recovered from Feedlot Cattle and Associations with Antimicrobial Use
Source: PLoS One. 2015 Dec 3;10(12):e0143995. doi: 10.1371/journal.pone.0143995 (PMC4669080; doi:10.1371/journal.pone.0143995)
Supplement: S2 Table — (DOCX) [file pone.0143995.s002.docx]

**Table S2.** **Interpretive criteria for *E. coli* using broth microdilution susceptibility testing reported as minimum inhibitory concentrations (µg/ml)**

| **Antimicrobial** | **Susceptible** | **Intermediate** | **Resistant** | **Reference** |
| --- | --- | --- | --- | --- |
|  |  |  |  |  |
| Amikacin | ≤16 | 32 | ≥64 | CLSI M100-S22, 2012 |
| Ampicillin | ≤8 | 16 | ≥32 | CLSI M100-S22, 2012 |
| Amoxicillin-Clavulanate | ≤8/4 | 16/8 | ≥32/16 | CLSI M100-S22, 2012 |
| Cefoxitin | ≤8 | 16 | ≥32 | CLSI M100-S22, 2012 |
| Ceftiofur | ≤2 | 4 | ≥8 | CLSI M31-A4, 2013 |
| Ceftriaxone | ≤1 | 2 | ≥4 | CLSI M100-S22, 2012 |
| Chloramphenicol | ≤8 | 16 | ≥32 | CLSI M100-S22, 2012 |
| Ciprofloxacin | ≤1 | 2 | ≥4 | CLSI M100-S21, 2011 |
| Gentamicin | ≤4 | 8 | ≥16 | CLSI M100-S22, 2012 |
| Kanamycin | ≤16 | 32 | ≥64 | CLSI M100-S22, 2012 |
| Nalidixic Acid | ≤16 | - | ≥32 | CLSI M100-S22, 2012 |
| Streptomycin | ≤32 | - | ≥64 | NARMS Exec. Rep. 2009 |
| Sulfisoxazole | ≤256 | - | ≥512 | CLSI M100-S22, 2012 |
| Tetracycline | ≤4 | 8 | ≥16 | CLSI M100-S22, 2012 |
| Trimethoprim-Sulfamethoxazole | ≤2/38 | - | ≥4/76 | CLSI M100-S22, 2012 |
|  |  |  |  |  |

CLSI = Clinical and Laboratory Standards Institute
