# Supplementary material for: Antimicrobial Resistance in Escherichia coli Recovered from Feedlot Cattle and Associations with Antimicrobial Use
Source: PLoS One. 2015 Dec 3;10(12):e0143995. doi: 10.1371/journal.pone.0143995 (PMC4669080; doi:10.1371/journal.pone.0143995)
Supplement: S3 Table — (DOCX) [file pone.0143995.s003.docx]

**Table S3.** **Interpretive criteria for *E. coli* using disk diffusion susceptibility testing reported as inhibition zone diameters (mm)**

| **Antimicrobial** | **[Disk] (μg)** | **Susceptible** | **Intermediate** | **Resistant** | **Reference** |
| --- | --- | --- | --- | --- | --- |
|  |  |  |  |  |  |
| Ampicillin | 10 | ≥17 | 14-16 | ≤13 | CLSI M100-S22, 2012 |
| Amoxicillin-Clavulanate | 20/10 | ≥18 | 14-17 | ≤13 | CLSI M100-S22, 2012 |
| Ceftazidine | 30 | ≥18 | 15-17 | ≤14 | CLSI M100-S18, 2008 |
| Ceftiofur | 30 | ≥21 | 18-20 | ≤17 | CLSI M31-A4, 2013 |
| Enrofloxacin | 5 | ≥21 | 17-20 | ≤16 | *CLSI M31-A4, 2013 |
| Florfenicol | 30 | ≥19 | 15-18 | ≤14 | *CLSI M31- A4, 2013 |
| Neomycin | 30 | ≥17 | - | ≤12 | *CLSI M31-A3, 2008 |
| Streptomycin | 10 | ≥15 | 12-14 | ≤11 | CLSI M100-S18, 2008 |
| Sulfisoxazole | 300 | ≥17 | 13-16 | ≤12 | CLSI M100-S22, 2012 |
| Tetracycline | 30 | ≥15 | 12-14 | ≤11 | CLSI M31-A4, 2013 |
| Trimethoprim-Sulfamethoxazole | 1.25/23.75 | ≥16 | 11-15 | ≤10 | CLSI M100-S22, 2012 |
|  |  |  |  |  |  |

* Interpretive criteria for *M. haemolytica* were used in lieu of *E. coli* as CLSI does not define breakpoints for *E. coli* in cattle for these antimicrobial drugs. CLSI = Clinical and Laboratory Standards Institute
